# Evaluation of embolization for periuterine varices involving chronic pelvic pain secondary to pelvic congestion syndrome

**DOI:** 10.6061/clinics/2016(12)05

**Published:** 2016-12

**Authors:** Flavio Meirelles Siqueira, Lucas Moretti Monsignore, Julio Cesar Rosa-e-Silva, Omero Benedicto Poli-Neto, Luis Henrique de Castro-Afonso, Guilherme Seizem Nakiri, Valdair Francisco Muglia, Daniel Giansante Abud

**Affiliations:** I Departamento de Medicina Interna, Divisão de Radiologia Intervencionista, Hospital das Clínicas da Faculdade de Medicina de Ribeirão Preto, Universidade de São Paulo; II Departamento de Ginecologia e Obstetricia, Hospital das Clínicas da Faculdade de Medicina de Ribeirão Preto, Universidade de São Paulo; III Departamento de Medicina Interna, Divisão de Radiologia, Ribeirão Preto/SP, Universidade de São Paulo, BrazilBrazil

**Keywords:** Chronic Pelvic Pain, Pelvic Congestion Syndrome, Embolization, Endovascular Procedures

## Abstract

**OBJECTIVES:**

To evaluate the clinical response and success rate after periuterine varices embolization in patients with chronic pelvic pain secondary to pelvic congestion syndrome and to report the safety of endovascular treatment and its rate of complications.

**METHODS:**

Retrospective cohort of patients undergoing endovascular treatment of pelvic congestion syndrome in our department from January 2012 to November 2015. Data were analyzed based on patient background, imaging findings, embolized veins, rate of complications, and clinical response as indicated by the visual analog pain scale.

**RESULTS:**

We performed periuterine varices embolization in 22 patients during the study, four of which required a second embolization. Seventeen patients reported a reduction in pelvic pain after the first embolization and three patients reported a reduction in pelvic pain after the second embolization. Minor complications were observed in our patients, such as postural hypotension, postoperative pain, and venous perforation during the procedure, without clinical repercussion.

**CONCLUSION:**

Periuterine varices embolization in patients with chronic pelvic pain secondary to pelvic congestion syndrome appears to be an effective and safe technique.

## INTRODUCTION

Chronic pelvic pain (CPP) is defined as frequent, non-cyclical pelvic pain of at least 6 months duration and affects approximately one-third of patients seeking gynecological care. CPP has an extremely negative impact on quality of life 1,2.

Pelvic congestion syndrome (PCS) is caused by pelvic venous insufficiency with retrograde flow in incompetent veins, usually the left ovarian vein, and development of pelvic varicosities 1. Similar to varicose veins in the lower limbs, these varicosities are due to a combination of incompetent venous valves, retrograde blood flow, and venous dilation. The symptoms are understood as a result of venous filling of varicose veins by a gravitational effect, and classical clinical presentations are pelvic or back pain that tends to worsen with standing and physical activity or in the late afternoon and is exacerbated before periods and after sexual intercourse 3. Pelvic varicosities and CPP are hallmark features of PCS, but women diagnosed with pelvic varicose veins can be asymptomatic 4.

The disease affects primarily multiparous patients of reproductive age for whom other causes of chronic pain must be excluded, such as infections, endometriosis, atypical menstrual pain, pelvic postoperative adhesions, pelvic inflammatory disease, and urological, musculoskeletal, psychiatric, and intestinal disorders 5.

In PCS, we find insufficiency in the left ovarian vein, presumably because its junction with the left renal vein forms a right angle, facilitating reflux. There are associations with mechanical compression, such as nutcracker and May-Thurner syndromes 7,8. The diagnosis of pelvic venous insufficiency is made by imaging studies, such as pelvic ultrasound with Doppler, pelvic computed tomography (CT), pelvic magnetic resonance (MR), laparoscopy, and transcutaneous venography. The latter is considered the gold standard in this field 9,10,11.

There are several treatment modalities for PCS, including drug therapy 7, surgical options such as hysterectomy with unilateral or bilateral oophorectomy and vein ligation, and endovascular treatment 11,12.

A few decades ago, endovascular treatment was proposed as an alternative treatment for PCS. The technique consists of accessing the pelvic veins and identifying the venous insufficiency by venography; then, embolization materials (metal coils or sclerosing agents) are placed to cause permanent occlusion and thrombosis of the territory. After occlusion of the insufficient veins, blood flow is diverted by the pelvic anastomotic network to drain the remaining veins, usually for the internal iliac veins. Endovascular treatment is minimally invasive and is performed under local anesthesia, thus enabling faster recovery for patients and shorter hospital stays 11,13,17. Despite the widespread use of the endovascular technique, published studies have used small samples with extensive technical variability and varying relationships between factors involved in the success or failure of clinical response.

Additionally, the clinical benefits of endovascular treatment have not yet been demonstrated in the Brazilian population. Thus, the aim of this study was to evaluate the efficacy and safety of endovascular treatment of PCS in a Brazilian medical institution.

## MATERIALS AND METHODS

This was a retrospective cohort from one center, with data evaluation of women treated in our medical institution. Data were analyzed in patients with CPP secondary to PCS who underwent endovascular treatment from January 2012 to November 2015, with a minimum follow-up of three months until March 2016. Patients with clinical suspicion of PCS who underwent venography without venous insufficiency and thus did not undergo embolization were excluded.

The following information was collected: age at diagnosis, time of symptoms, parity, history of pelvic surgeries, periuterine vein diameters visualized on MR before and after the procedure, reflux parameters in time-resolved magnetic resonance angiography (TR-MRA), and diagnostic venography, including embolized veins at the procedure.

**Figure 1:**
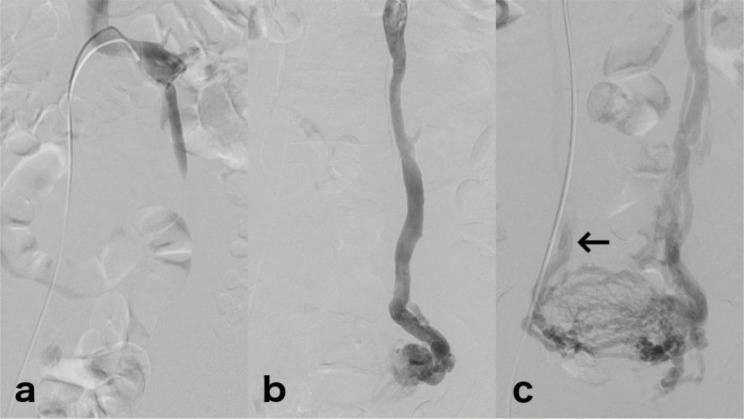
Venography of three different patients, exemplifying reflux grades. (a) Selective left renal venography shows confined reflux in the left ovarian vein corresponding to grade I reflux. (b) Left ovarian venography shows reflux in the dilated left ovarian vein and periuterine veins corresponding to grade II reflux. (c) Left ovarian venography shows a dilated left ovarian vein and periuterine reflux extending across the midline (grade III reflux). Note the contrast material reaching the contralateral ovarian vein (arrow).

The analysis of the TR-MRA and venographies involved the left ovarian vein reflux classification proposed by Hiromura in 2004 and was divided into three grades of reflux (Figure 1): grade I corresponding to confined reflux in the left ovarian vein, grade II corresponding to reflux reaching the ipsilateral periuterine venous plexus, and grade III corresponding to reflux crossing the midline and reaching the contralateral periuterine venous plexus 18.

Pain intensity was based on the visual analog pain scale (VAPS) and was assessed before and after the endovascular procedure, with reassessment after treatment (varying from 3 to 24 months). This scale was chosen due to its wide use for the clinical evaluation of pain syndromes 19. Technical success was defined as the absence of venous reflux at post-embolization venography, and clinical success was defined as pain reduction during follow-up.

In accordance with the multidisciplinary strategy of care at our institution, CPP patients in the Gynecology and Obstetrics service underwent detailed clinical and laboratory evaluations, including a physical exam, transvaginal ultrasound, urinalysis, urine culture and, less frequently, MR, urodynamics, cystoscopy, hysteroscopy and/or laparoscopy to highlight their main issues. Patients with CPP suspected to be secondary to CPS (with persistent pain even after drug treatment) were then referred to Interventional Radiology.

Patients with both clinical suspicion and limiting pain underwent venography regardless of confirmation of diagnosis by MR.

### Procedures

**Figure 2:**
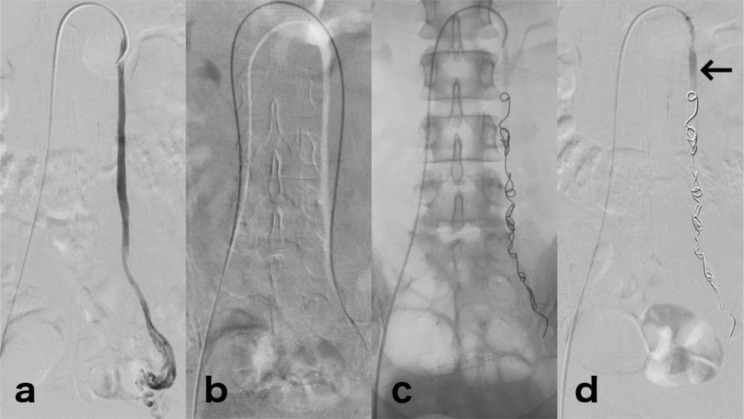
Embolization technique. (a) Venography pre-embolization with reflux in the left ovarian vein to periuterine veins. (b) Left ovarian vein catheterization to its distal segment using angiographic catheter over a hydrophilic guide wire. (c) Radiography after embolization, showing fibered metallic coils from the distal segment of the left ovarian vein to its proximal third. (d) Venography post-embolization, with complete occlusion of the vessel. Note the contrast material only in the proximal segment of the left ovarian vein (arrow).

All periuterine varices embolization (PVE) procedures were performed under conscious sedation and/or spinal anesthesia, without the use of heparin. The first embolization protocol consisted of right femoral vein puncture, followed by catheterization of the left ovarian vein with 5F diagnostic catheters (Simmons/Sidewinder 1, Cobra 1 or Cobra 2). When venous insufficiency of the left ovarian vein was confirmed, embolization of this vessel was performed with 0.035-inch fibered coils (VortX pushable Coils, Boston Scientific, USA) until occlusion occurred (Figure 2). The catheterization of other pelvic veins was not conducted unless left ovarian vein reflux was not present or another pelvic vein reflux was observed in previous TR-MRA.

Second embolization, if necessary, was performed by femoral or jugular vein puncture, followed by complete pelvic venography with the aid of diagnostic catheters 5F (vertebral, Simmons/ Sidewinder 1, Cobra 1 or Cobra 2). If an insufficient vein was present, we performed embolization with fibered coils until its occlusion.

After the procedure, patients remained in post-anesthesia observation and were discharged the same day or remained hospitalized for one day, depending on the time of the day the procedure was performed and how far the patient lived from the hospital. All patients were instructed about symptoms of pain during the first week post-procedure and were prescribed analgesics, nonsteroidal anti-inflammatories, and opioid drugs if necessary.

### Statistical analysis

Statistical analysis was performed by an independent, blinded statistician using IBM SPSS Statistics 20. Categorical variables are presented as numbers and percentages and were studied in terms of distribution frequency and compared among groups using the Chi-square or Fisher's exact test, as appropriate. Quantitative variables are presented as the mean, and the Mann-Whitney or Student's *t*-test was used as appropriate. A *p* value of less than 0.05 was defined as significant.

### Ethics

This study received approval from the local Research Ethics Committee.

## RESULTS

Twenty-seven patients underwent venography from January 2012 to November 2015, and PVE was performed in 22 patients. Five patients were excluded because they did not show venous insufficiency at venography and thus PVE was not performed. The age at treatment ranged from 29 to 55 years (mean 38.4 years, SD 6.9), with a time frame of symptoms from 6 to 216 months (mean 59.6 months, SD 48.6). Seven patients (31.8%) who underwent PVE were postmenopausal. Parity ranged from 0–7, with only one nulliparous patient. Fourteen patients had previous pelvic surgery, and 13 of those included Cesarean section. Laparoscopy (n=5), resection of endometriomas (n=2), salpingectomy (n=3), salpingo-oophorectomy (n=1), hysterectomy (n=1), tubal ligation (n=2), and bladder sling (n=1) summarize the cases of previous pelvic surgery.

Embolization of the left ovarian vein was performed in 21 patients (one patient underwent embolization of the internal iliac veins). In three cases, 2 veins were embolized during the procedure: the left internal iliac in one case, the right ovarian vein in another, and both internal iliac veins in the third case. Minor complications were observed in 4 cases, including 2 cases of venous rupture without symptoms or rebound hemoglobin levels, a case of limiting pelvic pain until the eighth postoperative day, and one case of postural hypotension in the first postoperative day. No cases of coil migration, bleeding with hemodynamic repercussion, contrast reactions, or death were reported. Individual patient data are shown in Table 1.

A second embolization was performed in 4 patients, with embolization of both ovarian veins in 2 of these patients: the two internal iliac veins in 1 patient and only the right ovarian vein in the other. One of these patients had limiting pelvic pain until the 15th postoperative day. All procedures demonstrated technical success.

The VAPS average before the procedure was 8.4 and decreased to 5.2 after the procedure, with the evaluation time post-procedure ranging from 3 to 24 months (mean 10.2 months, median 6.0 months, SD 7.9). Symptoms improved in 17 cases (77.3%). In retreatment cases, three patients showed improvement in symptoms (75%).

One case of clinical failure showed marked improvement (VAPS from 10 to 1) after laparotomy with lysis of abdominopelvic adhesions. Another case of clinical success with PVE while maintaining significant symptoms showed improvement (VAPS went from 7 to 0) after laparoscopy, with excision of a rectovaginal septum endometrioma.

Pelvic MR was performed before the procedure in 21 of 22 patients, and the diameter of periuterine veins ranged from 3 to 10 mm (average 6.7 mm, SD 1.6). Post-treatment pelvic MR was performed in 13 patients at times ranging from 4 to 35 months after the procedure; one patient did not undergo MR before the procedure. The diameter of the periuterine veins in post-treatment MR ranged from 2 to 7 mm, with diameter reduction occurring in eight cases (66.7%). In 12 patients, measurement of periuterine veins (both pre- and post-embolization) by pelvic MR revealed that the average diameter of periuterine veins was 6.5 mm before PVE and 4.8 mm after PVE (*p*=0.015). Clinical and procedural data can be found in Table 2.

TR-MRA was performed in 20 patients before PVE, and incorrect technique was used in 4 cases (images were acquired only in the arterial phase). Of the remaining cases, 14 had positive findings for reflux in the left ovarian vein; the Hiromura reflux classification was grade I for 6 cases, grade II for 4 cases, and grade III for 4 cases. Venography (performed in all 22 patients) showed left ovarian vein reflux in 21 patients, which was grade I in 3 cases, grade II in 13 cases, and grade III in 5 cases. The correlation of the presence of left ovarian vein reflux between TR-MRA and venography was 87.5%, whereas Hiromura reflux grade compatibility in that vessel between the methods was observed only in 7 cases (50%). The imaging and procedural data are presented in Table 3.

Comparing reflux grade with the clinical response, the five cases with grade III left ovarian vein reflux on venography exhibited symptom improvement after endovascular treatment (clinical success of 100%), whereas symptom improvement was observed in only 11 of 16 cases with reflux ranging between I and II in the left ovarian vein (a clinical success rate of 68.7%), but this result was not significant (*p*=0.27).

## DISCUSSION

PCS is a pathological condition causing chronic pain that usually affects young patients, with a high incidence of anxiety, depression, and sexual disorders 1,2. Treatment of this condition greatly improves patient quality of life.

Treatment of CPP secondary to PCS can be accomplished with hormonal therapy, surgical procedures, or embolization. Medical management can be achieved with medroxyprogesterone acetate or analogues of gonadotropin-releasing hormone (goserelin acetate), resulting in improvement of symptoms for up to four months that is not sustained long-term 7. As a surgical alternative, hysterectomy with unilateral or bilateral oophorectomy was reported to improve symptoms in up to two-thirds of patients after one year of clinical follow-up 20, whereas other studies did not show significant pain reduction 17. Failure of symptom improvement after hysterectomy may be due to the rich anastomotic periuterine network, which makes it more difficult to complete resection during surgery. Surgical risks, sterilization, and unsatisfactory results after hysterectomy, associated with the availability of less invasive methods, have eliminated hysterectomy as the first option for treatment. In 2003, Chung and Huh compared three treatment options for CPP secondary to PCS (PVE; hysterectomy with bilateral oophorectomy and hormone replacement; and hysterectomy with unilateral oophorectomy), with clinical results statistically favorable to PVE 12. Venous ligation of one or both ovarian veins has also been proposed as a treatment 11 but is rarely performed. The endovascular technique, which involves a shorter procedure time and faster postoperative recovery, is preferable to that of venous ligation 11. Various embolizing materials are used in the endovascular technique, including liquid sclerosing agents and metallic coils, and no significant differences in clinical outcomes were observed among the embolizing materials 1,2,11,13,17. In our study, all embolizations were performed with fibered metallic coils.

In our institution, we performed a different treatment protocol from that frequently found in the literature. Whereas pre-embolization venography involves the study of two gonadal veins, including the study of the internal iliac veins 1,2,7,13-16,21,23, the left ovarian vein was studied in patients with CPP who submitted to venography in our institution. The catheterization of other pelvic veins was not conducted unless left ovarian vein reflux was not confirmed or another pelvic vein reflux was identified in previous TR-MRA. This approach was based on the increased incidence of reflux in the left ovarian vein and reduced radiation exposure and the use of contrast material.

The overall clinical success rate of 76.9% is consistent with values reported in the literature of 60 to 100% 1,2,7,13-17,21-23, with significant pain reduction indicated by VAPS along with a reduction in the average periuterine vein diameters as determined by MR. In two patients (9%), we identified a concomitant cause of CPP after the performance of PVE.

A tendency toward a better clinical response was observed in patients classified as having grade III reflux by the Hiromura classification; however, the result was not significant (*p*=0.27).

During the evaluation period, 4 of 22 (18.2%) patients required retreatment, with reflux identification in veins other than the left ovarian vein in the second embolization. Therefore, the retreatment rate was attributed to a simplified initial venography protocol.

Minor and transient complications such as postural hypotension, limiting postoperative pain and venous rupture during the procedure without clinical repercussions were identified in 18.2% of cases. There were no permanent complications or death, consistent with literature descriptions 1,2,7,13-17,21-23. Coil migration, described at a 1.6% rate in the literature, was not observed in this study.

Our study has several limitations. First, it was a retrospective observational study and included a small number of patients, which prevents additional statistical analysis. Second, in our study, patients experienced variability in access time to the VAPS. Finally, the adjuvant treatment of patients included heterogeneous use of antidepressants, anxiolytics, and hormonal therapy with progestin, which eventually could represent a bias for evaluation of clinical response. A multicenter controlled study must be performed to confirm our findings.

In conclusion, the findings of this study suggest that PVE in patients with CPP secondary to PCS is a feasible and effective method to improve CPP symptoms and can be considered a valid therapeutic strategy for this Brazilian subpopulation.

## AUTHOR CONTRIBUTIONS

Siqueira FM and Monsignore LM conceived the study, participated in its design, coordination, and data collection, and drafted the manuscript. Rosa-eSilva JC, Poli-Neto OB and Castro-Afonso LH participated in the study design, data collection and helped draft the manuscript regarding their fields of knowledge. Nakiri GS and Muglia VF participated in the study design, data collection and imaging review. Abud DG conceived the study, participated in its design and coordination, and helped draft the manuscript.

## Figures and Tables

**Table 1:** Individual characteristics of the patients.

Patient (n)	Age	Parity (n)	Previous pelvic surgeries	Time from symptoms to embolization (weeks)	VAPS before/after embolization	Time from embolization to VAPS (months)	Minor complications
1	29	1	N	24	10 / 4	24	N
2	37	2	Y	96	10 / 7	24	N
3	32	3	N	36	7 / 2	3	N
4	34	2	Y	18	10 / 3	6	N
5	41	3	N	84	4 / 1	24	N
6	37	7	Y	36	9 / 7	6	N
7	55	4	Y	84	8 / 4	3	N
8	46	2	Y	48	10 / 8	6	N
9	38	2	Y	60	7 / 4	24	N
10	30	1	Y	72	9 / 9	9	N
11	48	3	Y	48	8 / 6	10	N
12	45	1	Y	36	9 / 0	6	Y
13	42	1	Y	16	7 / 6	7	N
14	44	1	Y	156	9 / 2	24	Y
15	34	2	Y	6	8 / 10	3	N
16	29	2	Y	216	10 / 1	6	N
17	37	2	N	24	6 / 5	6	N
18	35	5	N	36	10 / 10	10	Y
19	34	0	N	84	8 / 1	6	Y
20	30	1	N	36	9 / 6	3	N
21	44	2	Y	36	7 / 8	9	N
22	44	3	N	60	10 / 10	6	N

**Legend:** **(VAPS)** Visual analog pain scale; **(Y)** Yes; **(N)** No.

**Table 2:** Clinical and procedural data.

	Results
	**n=22**
Time from symptoms to embolization (weeks) (mean, range, SD)	59.6 (6-216, 48.6)
VAPS before embolization (mean, range, SD)	8.4 (4-10, 1.6)
VAPS after embolization (mean, range, SD)	5.2 (0-10, 3.2)
Time from embolization to VAPS (months) (mean, range, SD)	10.2 (3-24, 7.9)
Symptom improvement after embolization (n, %)	17 (77.3)
Minor complications (n, %)	4 (18.2)
	**n=21**
Diameters of periuterine veins (mm) on MR before embolization (mean, range, SD)	6.7 (3-10, 1.6)
	**n=13**
Diameters of periuterine veins (mm) on MR after embolization (mean, range, SD)	4.9 (2-7, 1.5)
	**n=12**
Reduction of periuterine vein diameter after embolization (n, %)	8 (66.7)

**Legend: (VAPS)** Visual analog pain scale; **(SD)** Standard deviation; **(MR)** Magnetic resonance; **(N)** Patient number.

**Table 3:** Patient radiological and procedural data.

Patients (n)	Periuterine vein diameter before/after embolization (mm)	Reflux grade of the left ovarian vein based on TR-MRA/venography*	Embolization sessions (n)/Veins occluded (n)	Veins occluded	Symptom improvement after embolization
1	6 / 6	NA / 0	1 / 2	RIV, LIV	Y
2	8 / 4	III / III	1 / 1	LOV	Y
3	8 / 7	I / II	1 / 1	LOV	Y
4	7 / 3	III / III	1 / 1	LOV	Y
5	10 / 6	NA / II	1 / 1	LOV	Y
6	7 / 4	NA / II	2 / 2	LOV, ROV	Y
7	8 / NA	I / II	2 / 2	LOV, ROV	Y
8	7 / NA	I / III	1 / 1	LOV	Y
9	5 / 6	III / II	1 / 1	LOV	Y
10	3 / 4	II / II	1 / 1	LOV	N
11	7 / NA	NA / II	1 / 1	LOV	Y
12	5 / 2	NA / II	1 / 1	LOV	Y
13	5 / NA	II / II	1 / 1	LOV	Y
14	6 / NA	I / I	1 / 1	LOV	Y
15	9 / NA	NA / II	1 / 1	LOV	N
16	5 / NA	I / II	1 / 1	LOV	Y
17	NA / 5	NA / III	2 / 3	LOV, RIV, LIV	Y
18	5 / 5	I / II	2 / 2	LOV, ROV	N
19	7 / 5	III / III	1 / 1	LOV	Y
20	8 / 7	0 / I	1 / 2	LOV, ROV	Y
21	8 / NA	II / II	1 / 2	LOV, LIV	N
22	8 / NA	II / I	1 / 1	LOV	N

**Legend:** **(NA)** Not available; **(0)** absence of reflux; **(TR-MRA)** Time-resolved magnetic resonance angiography; **(ROV)** Right ovarian vein; **(LOV)** Left ovarian vein; **(RIV)** Right iliac vein; **(LIV)** Left iliac vein; **(Y)** Yes; **(N)** No.

* Hiromura reflux classification.
